# Evolutionary decay and the prospects for long-term disease intervention using engineered insect vectors

**DOI:** 10.1093/emph/eov013

**Published:** 2015-07-08

**Authors:** J. J. Bull

**Affiliations:** ^1^Department of Integrative Biology;; ^2^Institute for Cellular and Molecular Biology; and; ^3^Center for Computational Biology and Bioinformatics, University of Texas, Austin, TX 78712, USA

## Abstract

After a long history of applying the sterile insect technique to suppress populations of disease vectors and agricultural pests, there is growing interest in using genetic engineering both to improve old methods and to enable new methods. The two goals of interventions are to suppress populations, possibly eradicating a species altogether, or to abolish the vector’s competence to transmit a parasite. New methods enabled by genetic engineering include the use of selfish genes toward either goal as well as a variety of killer-rescue systems that could be used for vector competence reduction. This article reviews old and new methods with an emphasis on the potential for evolution of resistance to these strategies. Established methods of population suppression did not obviously face a problem from resistance evolution, but newer technologies might. Resistance to these newer interventions will often be mechanism-specific, and while it is too early to know where resistance evolution will become a problem, it is at least possible to propose properties of interventions that will be more or less effective in blocking resistance evolution.

## INTRODUCTION

The technology of genetic engineering has improved radically in the last few years. One major ramification is that we are on the precipice of a radically new approach to subduing and even eradicating some infectious diseases and agricultural pests. For parasites that are transmitted to humans by insect vectors, we can theoretically intervene genetically either to destroy the vector population’s ability to transmit the parasite or even to possibly wipe out the species altogether. Yet these genetic interventions will almost universally impose costs on the vector or parasite that favor evolution of counter measures, and this opposing evolution may lead to the demise of the intervention. Avoiding this evolution may be essential to the long-term success of disease suppression. In turn, an ability to predict the evolution of these engineered genomes or of parasite evolution in response to the engineering will be important to the success of the intervention.

This article reviews some major interventions that have been proposed and explains the bases for predicting pathways of evolution in response to that engineering. We are witnessing a small explosion of proposed methods that may feasibly be engineered for vector control. Yet many of the concepts are decades old, now being reformulated in the context of modern technology; others are new. Our ability to predict evolutionary responses is yet shallow, but we can begin to identify possible principles to use in that effort. Anticipating and blocking evolutionary decay of these strategies may well be the next frontier in this field. However, before delving into possible mechanisms of resistance evolution, a review of proposed methods is required.

## GENETIC MECHANISMS OF VECTOR INTERFERENCE

The maintenance of a vector-borne disease in human populations requires vector competence to acquire and transmit the agent plus a sufficient abundance of vectors to ensure that an infection in one person leads to an infection in another before the first infection dies out. Possible vector-driven interventions are thus to interfere with vector competence, vector abundance or vector ecology as it relates to parasite acquisition and transmission [1–7]. Nearly all emphasis to date is on reducing vector competence or abundance, and those two objectives will be the focus here. The genetic strategies for reducing vector competence often differ from those for reducing abundance, although the two objectives may use a common technology. Furthermore, the implications for evolutionary decay of an intervention are specific to each goal as well as to the genetic mechanisms employed to achieve that goal.

‘Competence reduction’ involves genetically transforming the vector population to block parasite acquisition or transmission while leaving the vector population largely intact. The transformation may introduce a new gene or modify/delete an existing one. In contrast, ‘suppressing’ the vector population involves reducing its numbers, either through outright killing individuals or suppressing birth rates. Some of the new genetic technologies can be used for either process: the vector population is genetically transformed, but depending on which genes are modified, the outcome is reduced competence or suppression.

### Mechanisms to force genes into populations

The key element in all plans is to drive novel genes or mutations into wild populations, and also—for the goal of competence reduction—to get those genes/mutations to persist at high frequency for many generations. Some methods of driving genes and mutations are old in concept, but many are new and nearly all are so improved by modern technology that they can be implemented with vastly increased probability of success. Five types of mechanisms are receiving the greatest attention.

#### Inundation

This approach is simply to release many laboratory-reared insects so they mate with the wild populations in sufficient numbers to affect offspring genotypes. Whatever genes are carried by the lab-reared insects are infused into the offspring of wild parents and possibly into the descendants of further generations. Inundation may be employed as the sole gene drive mechanism to suppress populations (as part of the classic sterile insect technique, [8]), it may be used as the sole gene drive mechanism for competence reduction [9], or it may be used in conjunction with other mechanisms to drive genes into populations. If it is the sole mechanism used for population suppression, it must be applied continually to maintain the suppression, at least until extinction. The harmful genetic effect on the target species may be caused by single genes or mutations, clusters of genes, chromosome rearrangements, irradiated genomes or bacterial symbionts.

All other systems below have the advantage that gene frequency increases are driven at least in part by selective processes that have an intrinsic ability to cause spread with just limited inundation, perhaps as little as a few, genetically modified individuals.

#### Killer-rescue systems

First identified in bacteria [10], the combination of a gene that kills and another gene that rescues has been proposed in several designs that can potentially be implemented in insects. Both genes are introduced into the population: the rescue gene is protected from killing, but its ‘competitor’ is sensitive, so increase of the killer leads to replacement of the sensitive genotype by the rescue. As killer and rescue both become common, they reinforce each other’s advantage, the killer providing a selective benefit for the rescue, and the rescue protecting the killer from loss [11]. The killer and rescue genes may be tightly linked or be unlinked (see below). Their main use seems to be for vector competence reduction because their effect on population size is only temporary.

The ‘medea’ element operates in this fashion [12]. An otherwise lethal, maternally expressed effect—the killer—is introduced into all of a mother’s ova. Her offspring die unless they receive the rescue element (also encoded by ‘medea’) from either parent. Half the offspring of a heterozygous mother die if she mates with a male lacking ‘medea’; a fourth die if she mates with a heterozygous male. ‘Medea’ also experiences a transmission advantage if brood size is compensated for dead offspring, but its advantage otherwise depends on ‘medea’ being at sufficient abundance in the population to effect paternal rescue of offspring. (Brood size compensation is an increase in number of offspring or offspring fitness in response to inviability within the brood such that resulting combined progeny fitness offsets some of the lost siblings.) Akbari *et al.*. [13] engineered this type of element using a ‘killer’ miRNA that suppressed an essential, maternally expressed gene combined with a zygotically expressed rescue that consisted of the essential gene under embryonic expression. The construct operated as expected in *Drosophila* but has not been reported to operate in mosquitoes.

Gould *et al.*. [14] proposed a system in which killer and rescue genes are unlinked; both are introduced by inundation, but once present at moderate frequency, they reinforce each other despite the absence of linkage. If there is no fitness cost of either gene, the population can maintain the combination indefinitely once the rescue gene is fixed. With a fitness cost, the system will decay on a predictable schedule. This decay has the benefit of enabling the eventual reversal of the engineering, should some undesirable outcome emerge.

#### Heterozygote transmission advantage

Also known as ‘meiotic drive’, this is the classic selfish gene—in a heterozygote, the gene is transmitted to more than half the offspring. Segregation distorters, gene conversion systems, transposons and homing endonuclease genes (HEGs) have these properties, and HEGs have recently been engineered using the CRISPR/Cas-9 system (see below). With brood size compensation for dead embryos, the aforementioned ‘medea’ element [12] also experiences a transmission advantage [15]. Although meiotic drive systems were first entertained as vehicles to transform populations [2], they can also be used for population suppression [7, 16, 17]. Selfish genes are discussed more fully below.

Burt [7] sketched a hybrid system that combines killer-rescue with the transmission advantage of a HEG. The HEG is targeted to destroy the wild-type allele of an essential gene; its transmission advantage allows the HEG to spread through its selfish advantage. However, an engineered, insensitive rescue allele is introduced opposite the HEG and spreads both because of its fitness rescue and because it resists the HEG. Whereas the killer-rescue of Gould *et al.*. [14] requires substantial inundation, the HEG-mediated killer rescue needs little or none.

#### Heterozygote inferiority (underdominant fitness)

In this mechanism, natural selection operates against the least common of two (or more) alleles in the population, favoring the more common. This mechanism is suitable for introducing genes that interfere with vector competence but have only a small effect on individual fitness in the homozygous state. The most straightforward biology to achieve heterozygote inferiority uses chromosome rearrangements that lead to unbalanced gametes in the heterozygote [5, 6], now feasibly engineered [18, 19]. A system with heterozygote inferiority must be introduced by inundation or other forceful means to exceed approximately half of the total population, at which point natural selection will complete the sweep to fixation and maintain it (some versions require much less inundation than 50%, [20, 21]). Thus selection completes the drive beyond the initial inundation. Any genes completely linked to the new chromosomal morph will be fixed as well. At this endpoint, the effect of the engineered type on population numbers will often be slight or non-existent, depending on fitness of the new homozygote. Several new schemes proposed for engineering underdominance do not rely on chromosome rearrangements but use what might be called reciprocal killer-rescue: there are two different engineered segments, each carrying its own killer and carrying the rescue for the opposite killer [20, 22]. Marshall and Hay [23] developed an underdominant killer-rescue that relies on different developmental timing of killer and rescue, described as an inverse of ‘medea’.

#### Wolbachia

This obligately intracellular bacterium is found in possibly over half of all insect species [24]. It can invade new species seemingly at will, spreading by interfering with host reproduction. The fact that this bacterium can spread throughout a species from a small introduction renders it similar to selfish genes. Its self-promotion mechanisms commonly use a type of killer-rescue. Once established in the population, *Wolbachia* typically has minimal deleterious effects except in outcrosses, where it can effectively enforce speciation (males born of a mother with *Wolbachia* cause sterility when mated to a female lacking that strain of the bacterium). It can be used either to modify vector competence or for population suppression, but lasting suppression requires continual inundation [25]. At present, this bacterium is unsuitable for genetic engineering, but some strains have intrinsic properties that are useful for competence reduction—they block or inhibit the transmission of many viruses from its insect host [26].

There is a range of successes so far obtained with these methods, spanning actual field implementations proceeding as expected (the classic sterile insect technique, *Wolbachia* introductions), to success in cage populations (‘medea’ and inverse ‘medea’, CRISPR-mediated HEGs, Y chromosome segregation distortion) to almost no attempt at implementations, to outright failures (many sterile insect attempts); the following text will elaborate on several of these examples. The purpose here is not to suggest that all these methods will invariably work as advertised, but only that some of them will work well enough to set the stage for evolution of resistance and counter measures—the emphasis of this review.

## EXAMPLES OF POPULATION SUPPRESSION AND THE POTENTIAL FOR EVOLUTIONARY ESCAPE

It is well appreciated that, up to the point of extinction, population suppression imposes strong selection for escape. Selection does not imply evolution, however. As an example, virtually all population suppression mechanisms implemented to date in the classic sterile insect technique rely on mating. To the extent that a high degree of suppression is achieved, selection is intensely strong for many forms of asexual reproduction (parthenogenesis), sib mating or mating discrimination [27]. Yet successful escapes have rarely been reported as the basis of failure [28–30], even though mating discrimination has evolved in other contexts (e.g. [31]) and evolved once in response to the sterile insect technique [32]. Few species are perhaps capable of evolving asexual reproduction, but sib mating and mate discrimination should be accessible. The lack of resistance evolution from prior implementations of sterile insect inundation may thus stem from a combination of mutation insufficiency, rapid selection and inappropriate population structure [27]. For example, if population suppression leads to a local dynamic sink, migration inward from populations not subjected to the suppression may swamp local adaptation. Likewise, a rapidly shrinking population during treatment will progressively experience fewer mutations for an adaptive response, and those mutations that do arise may not be sufficient to persist amid further inundation. Beyond these general considerations, the nature of selection depends heavily on the genetic mechanism of suppression. Evolution of escape is intrinsically more plausible for some than for others.

In much of what follows, the potential for a population to escape—to evolve resistance to—an intervention is emphasized. It needs to be acknowledged that the mere fact that resistance ultimately evolves is not necessarily a reason for a method to be abandoned. Temporary population suppression or competence reduction may have a profound effect on disease incidence, possibly even allowing local parasite extinction. Even short-lived local population suppression may have a lasting effect on population size, depending on migration from other populations and the speed with which the population rebounds from surviving foci.

### Inundation and the sterile insect technique: background

The concept of genetic suppression of populations is old, approaching 8 decades, with independent proposals from Serebrovsky in Russia [33], Vanderplank in Africa [1] and Knipling in the USA [3, 4]; an excellent review is that of Klassen and Curtis [8]. The most famous implementation was eradication of the screw worm from North America, a feat developed and spearheaded by Knipling and Bushland (references above). The method—the sterile insect technique—relies entirely on inundation but otherwise seems simple and widely generalizable: (i) laboratory-reared individuals are irradiated to the point of gamete damage but not behavioral impairment, (ii) they are released at sufficient densities so that irradiated individuals dominate matings of wild individuals, (iii) nearly all offspring of those wild individuals die from the mate’s defective contribution and (iv) the population size declines. Each decline is followed by additional releases, and the population ratchets down further; suppression occurs more easily at each step because the decreasing abundance of wild individuals is increasingly overwhelmed by the steriles. The releases must be continued indefinitely or until the population disappears. In many applications, the releases are just of sterile ‘males’, but in some cases, steriles of both sexes may be released.

The earliest proposals for the sterile insect technique relied on using the natural sterility between chromosome races [33] or the sterility between closely related species, which often had an unknown basis [1]. The bacterial symbiont *Wolbachia* may sometimes have been the basis of inter-strain sterility [34, 35]. The use of radiation to induce sterility was Knipling’s idea and was inspired by the attention drawn to Muller’s work on radiation after the 1946 Nobel Prize (described in reference [8]). Once the sterile insect technique gained recognition, various other creative methods of sterile induction were attempted, including insect traps that dabbed mutagenic chemicals on the insect before it flew off [36, 37]. Some methods required the release of lab reared individuals (radiation or chromosome rearrangements), some operated entirely in the wild (traps with chemicals).

The sterile insect technique has been attempted many times against a variety of species [28, 30, 37]. There are many successes, many partial successes in which some degree of population suppression was achieved, and many failures [8, 38]. Initial successes were not always pursued. The challenges were and are many, from biological and ecological [39, 40] to political. (i) Radiation does not always work, as it may not be possible to find a dose for which most sperm genomes are destroyed but sperm retain the ability to fertilize and male mating behavior is still normal. (ii) For methods that relied on chromosomal rearrangements, the rearrangements needed to be generated in the lab and often failed because of background fitness effects induced by the mutagenic methods used to generate the rearrangements. (iii) For some applications, only males can be released because sterile females would cause damage (e.g. transmit disease); some applications were prohibited for lack of a means to selectively isolate males from lab populations. (iv) In at least one case, the lab population adapted to the artificial rearing conditions in ways that made it less competitive in the field [41]. Problems have sometimes been political, preventing the continual or wide-scale release of the steriles.

It is also appreciated that ecology is critical in the sterile insect technique [8, 33, 39]. Importantly, enough sterility-inducing insects need be released to overwhelm matings in the wild. Thus the sterile insect technique is best applied to species at low densities with low migration rates, as at the leading edge of species ranges or on islands. Indeed, migration was the basis of failure in some early applications to mosquitoes [29]. It is also appreciated that killing embryos is not necessarily as effective as killing older life stages, because density-dependent regulation at later stages may largely compensate for embryonic death [42]. However, density dependence presumably becomes less important after the initial decline in population size.

Of the many implementations of the sterile insect technique that outright failed or were not pursued, it is interesting that there seem to be few documented failures from evolution of resistance [27], an exception being McInnis *et al.*. [32]. Given that many efforts have been ongoing, such that surviving populations are continually selected for resistance, the lack of resistance is remarkable. It may well be that resistance was an undocumented contributing factor to failure or was too slight to be detected. However the lack of resistance bodes well for newer implementations using genetic engineering. At the same time, genetic engineering presents new opportunities for resistance evolution that would not have been favored against these older methods.

### Genetic engineering: prospects for long-term suppression by inundation

#### Dominant lethal

An exciting, new genetic implementation of the sterile insect technique is the engineering of dominant lethal genes. Insects bearing the dominant lethal are reared with the lethal unexpressed (suppressed by compounds in the lab food), they are released in the wild, mate, and their progeny express the lethal and die [43]. There are obvious advantages. Careful engineering minimizes any impact on the mating behavior of lab-reared individuals and can be facilely introduced to new strains [44] The age and sex of death is also easily controlled and can be delayed until the pupal stage, a potential ecological benefit over techniques that kill at the zygotic or embryonic stage [45].

A separate dominant lethal approach, anticipated by Burt [7], is afforded by the fortuitous discovery that the ribosomal gene cluster is on the X chromosome of *Anopheles* mosquitoes and that an endonuclease from a slime mold cuts a sequence in the ribosomal repeat [46]. With versions of the endonuclease protein engineered to be unstable, mosquitoes that express the endonuclease in spermatogenesis produce nearly all sons because X-bearing sperm have a broken X and lead to dead embryos. In contrast, use of the stable, wild-type enzyme results in complete sterility of the male because enzyme is transmitted through sperm and destroys the maternal X of embryos. Both designs provide alternatives to radiation as a means of producing sterile males, with the obvious advantage that male behavior would be unaffected. That one form of these ‘sterile’, engineered males produces only sons is, if anything, ecologically advantageous over complete sterility [7].

With the promise of easier applications from genetic engineering, what is the potential for evolution of escape mechanisms? With radiation-induced sterility, the only conceivable evolutionary pathway to escape (aside from asexuality) is for wild females to selectively mate with wild males [27, 47, 48]; there is no known zygotic mechanism of repairing multiply broken chromosomes from the sperm. Even if assortative mating begins to evolve, increasing the inundation may overcome the effect [27].

In contrast to the expected longevity of radiation-induced sterility, engineered systems whose lethal effect comes from gene expression after fertilization could have a shorter lifespan of utility [48]. These new methods require gene expression in the embryo to effect lethality, providing a new avenue of resistance. Escape may thus be simply a matter of suppressing lethal gene expression in the progeny, possibly an interfering or micro RNA or a mechanism already in place used to suppress transposable element activity. Resistance would need to be expressed from the maternal genome and provide enough of a selective boost to overcome the influx of sensitive genomes from the inundation and from migrants of untreated populations. However selection imposed by mortality is potentially strong enough to overcome these opposing processes. Whether such resistance is mutationally feasible is unknown. If resistance evolution proves to be a problem, designs that destroy genes pre-zygotically may be more stable, as considered next.

The prospects for resistance evolution against the aforementioned ribosomal-targeting endonuclease are different, because the lab-reared males have already destroyed the X chromosome in their sperm before they encounter wild females. In this respect, the method parallels radiation-induced sterility. There is thus no opportunity for selection in the wild to reverse this destruction. (Even a suppressor mutation that arose in the lab population would have no benefit in the wild.) Mating discrimination by wild females offers one escape, just as with irradiated males; female production of XX ova might be an escape under some highly restrictive mechanisms. Nonetheless, the system seems protected from many types of resistance evolution.

#### Translocation heterozygotes

Inundation from strains with chromosomal rearrangements (chiefly balanced translocations) has long been appreciated to allow transient population suppression [5, 6, 33]. The suppression effect relies on mis-segregation of multiply paired chromosomes, and the magnitude of sterility increases with the number of rearranged chromosomes [33]. When introduced at an appropriate level, translocations seem to be an almost resistance-proof mechanism of maintaining population suppression because of the invariant regularity of meiosis (mating discrimination against the steriles is always a possible escape). There is of course the risk that too much inundation will fix the rearrangements, at which point the lab strain would need to be replaced with a different chromosomal race; this problem can be overcome by releasing the translocation heterozygotes themselves, which are already sterile. Evolution of balanced segregation as an escape mechanism seems unlikely, some natural systems of balanced translocations notwithstanding [49].

Engineering chromosomal translocations seems only to improve the prospects over older methods, which attained translocations haphazardly, often by irradiation. The background mutations that accompanied the haphazardly acquired translocations in older implementations [29] are now easily avoided with engineering [18, 19], such that translocation homozygotes can now be as fit as the wild-type. Engineering also allows precise targeting of translocation breakpoints to maximize deleterious effects in the heterozygote. The easy engineering of rearrangements should allow the generation of lab strains with many rearrangements, reducing the fraction of gametes from heterozygotes that happen to contain a proper balance of genes [33].

### Suppression by transmission-distorting selfish genes

Beginning with the earliest attempts [1], many apparently successful implementations of the sterile insect technique were abandoned for lack of a sustained effort or for political reasons [8]. The appeal of using a selfish gene, fully appreciated from the start, is that a single introduction from just a few genetically modified insects can lead to the gene’s rapid ascent and possible fixation in the population with no further human effort. With some implementations, the selfish gene’s ascent causes a decline in the population size, potentially to extinction. If selfish-gene imposed population suppression can avoid evolution of resistance, it has the potential to overcome the economic and political impediments that stalled many prior efforts.

#### 50-year precedents

In the first half of the 20th century, unusual genetic systems were discovered in wild populations: the T-locus of mice and *Segregation Distorter *of fruit flies [50–52]. Both systems exhibited high frequencies of chromosome regions that violated Mendel’s rules and displayed strong segregation distortion in males—the Aa heterozygote transmitted far greater than 50% of A in its gametes. Earlier, Gershenson [53] had discovered a *Drosophila* X that was transmitted at a higher rate than the Y (hence the sires produced mostly daughters), but it was the later discoveries that led people to realize the full implications. An allele with a transmission advantage would normally have spread to fixation rapidly and no longer have been detectable [54], but in these new cases the homozygotes for the distorting allele were lethal or sterile and so could not go to fixation. Normally, a lethal or sterile allele would be lost from the population. The combination of the segregation advantage and the lethality led to neither outcome—the allele was maintained polymorphic. Population genetic models of segregation distortion of a recessive lethal showed that 100% distortion in both sexes resulted in fixation of the lethal [55, 56], where as 100% distortion in just one sex led to pure heterozygotes and a consequent 50% loss of offspring [56, 57]; the distorting allele was maintained at progressively lower frequencies with less distortion. Fixation of a recessive lethal implies extinction, although those early papers addressed gene frequency evolution rather than demography.

The potential use of these distorting, selfish genes for insect control was realized almost immediately. Sandler and Novitski [2] suggested that distorters could be used to drag other, linked genes into populations. Hickey and Craig [16, 58] worked out the genetics of sex-linked genes overproducing males in mosquitoes and realized their potential as agents to reduce the reproductive output of mosquito populations. An especially clear, early model for population control with a selfish gene was by Hamilton [17], elaborating on the suggestion of Hickey and Craig [16, 58] to use sex-linked segregation distorters. Y chromosomes were selected toward ever-increasing segregation distortion, producing ever higher numbers of sons. Hamilton specifically proposed that ‘driving’ Y chromosomes vastly overproducing sons might be a mechanism for population extinction.

Within a decade of Hamilton’s article, experimental tests of extinction were underway [59–61]. Although those times predated synthetic biology, and indeed, predated easy DNA sequencing, Terry Lyttle used standard genetic tools to create a fruit fly (*Drosophila*) in which the normal Y chromosome was effectively fused with an autosome that exhibited extreme segregation distortion. The segregation distortion of an autosome had been discovered in the same lab (of J.F. Crow) nearly 2 decades earlier, and Lyttle used standard X-irradiation to create chromosome fusions at random. By crossing hundreds of flies, he found some males that produced only or mostly sons. He could then visualize their chromosomes under a microscope to understand which chromosomes were fused. The critical property for his test was that males produced mostly sons and that this distortion was inherited patrilineally—all sons and grandsons also produced nearly all sons.

**Figure 1.**
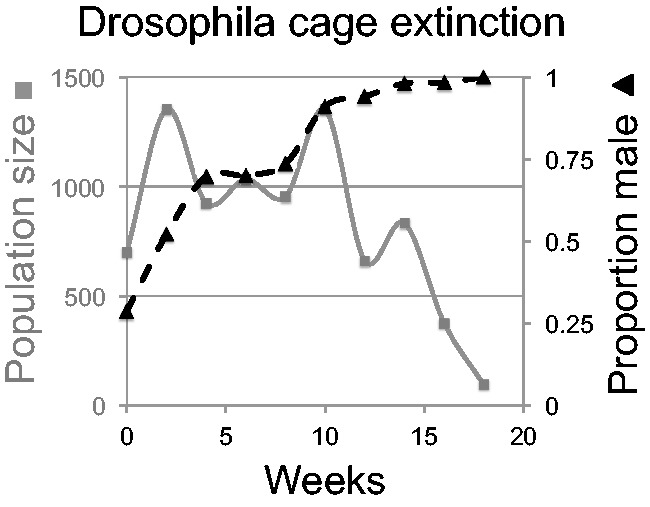
Dynamics of sex ratio and population size in an experimental *Drosophila* population started with 10% of males carrying a Y-linked segregation distorter producing nearly all sons. The population size appears to be unaffected until the population sex ratio (proportion male) becomes extreme; males were completely absent at the final sample. This is one of several replicates from Lyttle [59]; the horizontal scale uses the suggested 2-week interval between sampling

Several cage populations of flies were established with the distorting Y*. The population sizes were on the order of 1000 and the founding populations lacked known resistance alleles to the segregation distortion that occurred in the wild. In some, the selfish Y* spread to fixation with loss of females and thus extinction, as predicted (Figure 1). In other cages, the Y* had imperfect distortion, producing some daughters. Those populations also evolved fixation of Y* but without extinction; they then evolved a gradual decrease over time in the distortion due to the accumulation of weak suppressors. In one cage, however, the outcome was totally unexpected: the population evolved sex chromosome aneuploidy in which the presence/absence of Y* no longer determined sex (XXY* was female, XY* and XYY* were male). The evolution of this outcome is due to the specifics of *Drosophila* sex determination—the Y is largely void of genes and does not determine maleness *per se*, rather an individual becomes male if it has only a single X chromosome. Females were not lost from the population because the XXY* females produced some XX ova—which became female when fertilized with Y* sperm. However, the evolved aneuploid system was inefficient, and population fecundity suffered. Lyttle’s studies may have established a precedent for resistance evolution with other types of selfish gene population suppression.

#### Selfish genes causing extinction via gene destruction: homing endonuclease genes (HEGs)

Selfish genes may be used for suppression in other ways. In a truly insightful article that anticipated genetic engineering technology by more than a decade, Burt [7] proposed using HEGs for extinction as well as for competence reduction. HEGs have the property that a HEG heterozygote transmits as a homozygote—segregation distortion. The single copy of the HEG cuts the opposite chromosome at a specific site and inserts a copy of itself, so the heterozygote physically becomes a homozygote. Thus the HEG not only has a selfish advantage in transmission that enables its rapid fixation, but it destroys a specific sequence in doing so. If that target sequence is in a gene or promoter, the spread of the HEG is accompanied by destruction of the target gene or its expression. A protocol for engineering HEGs to insert at a sequence of choice has recently been published and promises to enable applying the technology to many species [62].

An unintuitive result is that the HEG will still spread even if it targets an essential gene, provided that a single copy of the essential gene in somatic tissues is sufficient for nearly normal fitness and that the HEG converts the heterozygote to a homozygote only in the germ line. This result is essentially the demographic consequence of earlier models of a recessive lethal with extreme segregation distortion [55, 57]. In simple cases, a HEG will spread if the heterozygote transmits the HEG to more total offspring than would equal half the total offspring produced by an average, wild-type individual. Thus if the survival of the HEG heterozygote is 3/4 that of the wild-type homozygote and the HEG is transmitted to 4/5 of the progeny, the net transmission of the HEG is 3/4×4/5=0.6, more than the 0.5 threshold for increase.

Even though a zygote dies when both parents contribute the HEG allele—such a zygote has no functional copies of the essential gene—the HEG can spread possibly to fixation, guaranteeing population extinction, provided its transmission in heterozygotes of both sexes is high enough. Complete fixation of a recessive lethal requires 100% transmission in both sexes, but population extinction need not require fixation. The equilibrium frequency of the HEG depends on the level of distortion in each sex and the fitness effects of the disrupted gene [7, 11, 63], but the generalization from earlier analyses still holds, that high levels of transmission distortion can overwhelm strongly debilitating fitness effects of the disrupted genes [55–57].

Selection does not stop at a single copy per genome of the HEG. Indeed, Burt [7] suggested ‘stacking’ multiple HEGs in a species to maximize population suppression and minimize evolution of resistance. It likewise follows that, if a single HEG could change genomic locations and simultaneously acquire the target sequence to that new location, it would have the potential to expand its genomic niche (subject to the fitness constraints for spread at that site). Were this kind of expansion possible, the invasion of one HEG into a population could lead to a selfish gene ‘cancer’ of the genome, with the HEG progressively jumping into new sites and continuing its expansion. Current HEG designs do not readily lend themselves to easily jumping and simultaneously acquiring the correct homing sequence, a possibly fortunate property that might ease concerns about releasing engineered HEGs. It is not inconceivable that such a system could be engineered, however.

##### Evolution of resistance to HEGs

Now that HEGs can be easily engineered and targeted to any chosen DNA sequence in many species, the technology would appear to have many useful applications, as anticipated by Burt [7]. Aside from concerns about the potential downsides of the technology [64], attention is likely to shift to mechanisms of resistance, since resistance can undermine the hoped-for utility. Insightful discussions of resistance and related issues are found in Burt [7, 65] and Esvelt *et al.*. [66].

From previous work, there are grounds for both optimism and pessimism about the potential evolutionary decay of these systems. On the positive side, Lyttle’s caged populations failed to fully overcome negative consequences of the driving Y. And neither the mouse t-allele nor the *Drosophila Segregation Distorter* has disappeared in the wild, despite their absolute sterility/lethality in homozygotes. On the negative side, Lyttle’s experiments exhibited diverse evolutionary mechanisms of resistance to a selfish gene in small, genetically homogenous populations. Likewise, it is discouraging that mice and *Drosophila* seem to experience no more than modest impact on species abundances from their segregating lethals. The observed frequency of mouse t-locus heterozygotes is even less than expected from the measured segregation ratios [67], suggesting that many factors may be mitigating the population suppression.

*A priori*, it is easily appreciated that any agent with the potential to eradicate an entire population will, at some point, face profoundly intense, opposing selection. Selfish genetic agents acting within genomes are no exception [68]. Resistance may come in many forms, and it can evolve even if fitness suffers profoundly, when the only alternative is zero fitness. Unless there are ready pathways for escape that entail no more than a slight drop in fitness (see below), predicting the details of resistance evolution will be nearly impossible except through experience. However, predicting that some form of resistance will evolve is much safer, hence Leigh’s generic concept of the ‘parliament of the genes’ as a way of acknowledging the many ways that evolution may lead to suppression of selfish genes [68].

Possible mechanisms of resistance can be divided into molecular and ecological. Molecular resistance to HEGs could be achieved by changes in the insert sequences, whether as natural polymorphisms or mutations created by the repair system [7, 66]. In this respect, the selection imposed by HEGs is similar to that imposed by restriction endonucleases and other sequence-based inhibitors [69, 70]. The suggested solution to avoid this resistance is merely to introduce enough, different HEGs at once to push the chance of multiple resistance near to zero [7], similar to the strategy of treating microbes with multiple drugs simultaneously to avoid the evolution of complete resistance. Resistance could also arise by blocking expression of the HEG or interfering with the HEG complex. Although there are many possible molecular mechanisms that might block expression, their feasibility is largely unknown, and outcomes may vary widely between species. However, the fact that meiotic sex chromosome inactivation spans insects to mammals [71, 72] suggests that there are mechanisms readily available to suppress gene expression in gametogenesis—perhaps the main tissue in which suppression of a HEG would be selected. Likewise, recently-discovered mechanisms of endogenous virus suppression also point to the potential of suppressing selfish genes [73]. Whatever the chances of resistance evolution by these mechanisms, a reasonable conjecture is that there will be more molecular mechanisms and thus more opportunities for resistance evolution if the HEG moves in both sexes than in just one sex. The benefit for a suppressor is increased progeny survival, which will be realized in whichever parents experience the homing. Offsetting this increased opportunity for resistance evolution when both sexes experience homing is the faster spread of the HEG (as pointed out by a reviewer).

Possible ecological mechanisms of resistance involve changes in mating behavior and life history. HEGs rely on heterozygotes for spread, and any mating structure or ecology that reduces heterozygotes will not only reduce the spread but may hasten the appearance of resistance. We may conjecture that asexuality, sib mating, other forms of inbreeding and group structure may all achieve various degrees of escape and may thus be expected to evolve [7, 66]. Maynard Smith’s haystack model of evolution of a selfish element with group structure provides a theoretical precedent illustrating how group structure can prevent fixation of a selfish element [74]. In plants, selfing would have much the same effect as asexuality, so success with HEGs might be limited to dioecious and self-incompatible systems. Theory is needed to address these and other possibilities, as the interaction between a HEG and genetic variation in mating structure is unintuitive.

Esvelt *et al.*. [66] raised the interesting possibility of using engineering to limit the spread of HEGs. Theirs is a specific proposal of designing an arms race among different engineered elements opposing each other’s fitness gains. The need to employ such counter strategies will depend on how easily natural populations intrinsically evolve resistance.

#### Sex-linked drive

As shown by Lyttle [59–61], selection imposed by sex-linked segregation distortion can favor extreme mechanisms of resistance. The recent success in engineering a nuclease-based destruction of the *Anopheles* X chromosome sets the stage for engineering a Y-linked drive element [7, 46]. Interestingly, that system seems more apt to avoid resistance evolution than many other possible Y-drive systems. The fact that the X chromosome is destroyed by cleavage of ribosomal gene sequences means that XY females would not be a favored escape (if the nuclease was also expressed in the female germ line). The abundance of target sequences on the X means that escape by incrementally changing each copy of the target sequence is unlikely [7, 46], although gene conversion in ribosomal clusters is known [75] and could lead to non-incremental changes in copy number.

### Evolutionary decay of competence reduction

The principle behind competence reduction is to leave a species numerically intact but alter its ability to transmit a disease agent. A major effort underway and pioneered by Scott O’Neill uses *Wolbachia* to prevent the mosquito *Aedes aegypti* from transmitting dengue virus and possibly other viruses [76]. It can be hoped that other implementations of competence reduction will soon follow, as genetic engineering provides many new ways of species-wide genome modification. The design might be to knock out a non-essential vector gene that the parasite requires for transmission or to carry in a novel gene that blocks transmission [7, 13, 77]. The design might be to change ecology so that the vector does not bite humans or to reduce vector lifespan slightly so that the parasite does not have time to mature [78, 79]. The fitness effect on the vector will typically be much lower than with population suppression. However, a new evolutionary problem arises with competence reduction that was absent with population suppression: selection may favor the parasite to overcome the block.

#### Vector resistance evolution

Any of the drive mechanisms listed at the front of this article may possibly be used to infuse genes for competence reduction. Long-term selection in the vector will be largely in response to the fitness effect of the transformed state, not the transformation process, because populations are transformed rapidly with most approaches, and when complete, there is little other impact on the population from the transformation process itself. However, because selection on the transformed state never ends—the transformed population persists indefinitely—seemingly small fitness effects can have large, long-term impacts. From basic population genetics theory [80], even a 10% fitness loss of the derived state would select return of the wild-type in just over 80 generations (from an initial frequency of 0.001 to 0.5, assuming complete dominance of the wild-type), yet a fitness effect of 10% is difficult to detect in short-term experiments. Thus, either the genetic nature of the transformation should be chosen carefully to minimize this fitness cost, or the mechanism of transformation should be chosen to block mutational reversion to the wild-type state. In this respect, destroying a gene with a homing endonuclease seems more prone to avoid reversal than does introducing a novel gene that could be inactivated by many types of mutations [7]. As a first step that might be used in this effort, work is underway to identify vector genes necessary for competence (e.g. [81–86]). Parasite-blocking variants that are segregating in the wild are likely to have low fitness costs and should be ideal candidates.

Some engineered approaches may lead to an outcome in which vector competence is blocked throughout much of the population for several years, but the entire population is not transformed [14]. The transformed state in these cases is prone to eventual decay by migration from adjacent, wild-type populations unless the transformed state has the higher fitness [87] or the transformation is driven by a selfish gene. Nonetheless, temporary success of a transformation may be sufficient to eradicate a disease, at least locally. And predictable senescence of a human-imposed genetic modification may be desirable from a regulatory perspective [14].

#### Parasite resistance evolution

The second serious concern is parasite evolution to overcome the block, an outcome that will avert eradication no matter how lasting and complete the population transformation may be. Whereas we have much experience with viral and other parasite evolution in response to drugs [88] and to vaccines, we have much less experience with parasite evolution in response to genetic variation in the host, especially for invertebrate hosts (but see [73], for vertebrates).

There is little work done on viral evolution to escape host factor modification in eukaryotes (the ΔCCR5 deletion in humans that blocks HIV infection being a noteworthy exception, [89]), but there are studies from bacteriophages, and those mostly point to the power of viral evolution [90]. Several ‘essential’ host factors were identified for the bacteriophage T7, but many were only partial blocks, and only one (*trxA*) proves insurmountable in the face of viral adaptation [91]; absence of *trxA* imposes a high cost on the host (Bull, unpublished work). The host protein rep (used in DNA metabolism) is required for replication by many phages [90]. Although full knockouts are apparently insurmountable, other *rep* mutations that provide initial blocks to ϕ X174 replication are overcome by a variety of point mutations [92]. The *Escherichia coli* receptor for phage lambda is considered essential for the virus, but prolonged adaptation of the virus led to use of a new receptor [93]; likewise, T-even phages have shown an ability to evolve use of new receptors [94]. This list is not complete. To deal with the challenge of engineering competence reduction that withstands parasite adaptation, experimental evolution of the parasite, perhaps in tissue culture, may be required to assess the ‘evolution-proof’ protection provided by a particular genetic block.

The blocking of viral transmission by some *Wolbachia* presents its own set of challenges to predicting evolutionary escape. The bacterium is so far recalcitrant to genetic manipulations, so its permanence of viral blocking and the possibility of viral evolution in response to blocking is a looming black box, providing little basis for prediction [95]. Although the *Wolbachia* approach may succeed, it provides much less latitude in tailoring the intervention to specific viral details or viral evolution.

### Evolutionary collapse of the engineering

An implementation may fail because the engineering is intrinsically unstable to evolution [96, 97]. For example, in many proposals for competence reduction, the mechanism of forcing genes into populations (such as killer-rescue) must be linked with a gene that causes competence reduction. Hitch-hiking with the forcing genes then causes the ascent of the competence reduction gene. Even rare recombination that separates the competence reduction gene from the forcing genes may limit the spread of competence reduction.

Instability is a property of most proposed engineered underdominant fitness designs using reciprocal killer-rescue systems (e.g. the designs of [20, 22]) and the ‘inverse medea’ element [23]. The heterozygote inferiority relies on a killer gene, and mutational loss of the killer provides an unconditional benefit until the rescue is fixed in the population (Hay, personal communication).

For killer-rescue systems that do not generate underdominance, the killer component may often be only neutrally stable or unstable. In the absence of brood size compensation, a mutant ‘medea’ element lacking the killer component has the same fitness as ‘medea’ (hence is approximately neutrally stable). Any cost to carrying the killer gives the killer-negative ‘medea’ the advantage; but because the rescue should increase regardless of whether it also carries a functional killer partner, the rescue could still be used to transform the population if the killer attains moderate frequency. The killer-rescue system of Gould *et al.*. [14] is in fact designed so that the killer is unstable up to the point that the rescue is fixed, whence killer becomes neutral; instability of the killer is overcome by inundation.

Engineered genes with transmission distortion are unusual in that they are expected to be self correcting toward increasing transmission bias, and this self-correction implies evolutionary stability [7, 17]. In contrast to other killer-rescue systems, the hybrid HEG × killer-rescue system of Burt [7] appears to be stable because the killing is a byproduct of (stable) HEG transmission distortion.

### Future: experimental tests, combined approaches

With the improved feasibility of genetically engineering vector populations, interest is likely to turn toward the evolutionary decay of those systems. Whereas *a priori* predictions were possible in the design of those systems, predicting their decay will be far more challenging, at least until we have more experience. That experience can come from actual implementations, of course, but it may be far more expedient to test the methods in experimental populations, as done by Lyttle with a distorting Y chromosome. If the implementation of one design in a wild population hinders the future use of other designs in that population, it will be important to use a well-informed implementation at first release.

There is yet little basis for predicting the detailed evolution of escape mechanisms for many of the proposed interventions. Yet the bases of escape from some mechanisms can be anticipated at a broad level, and others can be put forth as conjectures; many are yet vague. Some principles apply to population suppression, others to competence reduction, some to both:

Principles for both types of interference
  - Stacking agents will limit resistance evolution and increase efficacy.
  - Genetic engineering that imposes a fitness cost will be more prone to resistance evolution if expressed in both sexes than if expressed in just one sex.
  - Mechanisms that rely on inviolable biology (chromosome integrity, meiosis, fertilization) will be the most stable against escape.
  - Inbreeding and group structure slow the spread of harmful selfish elements and enhance evolution of resistance.

Principles specific to population suppression
  - With inundation to suppress populations, numbers should be depressed quickly and as completely as possible. Small population sizes are least prone to evolve resistance. Migration inward from unaffected populations may retard resistance evolution.
  - Sterility created prior to release of the insect is less prone to resistance evolution than is sterility or death resulting from processes expressed in the progeny of wild parents.
  - Sequence-specific mechanisms of population suppression are prone to predictable and easy means of escape by changes in the target sequence. Those pathways must be anticipated and blocked or overwhelmed.

Principles specific to competence reduction
  - Vector genes least prone to evolution of parasite escape are those conserved between related vectors and those required by related parasites.
  - Gene knockouts are less prone to evolutionary reversal than are introductions of functional genes.

In the final analysis, success in controlling vectors may be an arms race that requires combined approaches with frequent updates [7, 65, 66, 98, 99] An apparent benefit of genetic approaches over pesticides is that there are multiple methods that have little mechanistic overlap, hence the potential for combining them without cross resistance evolution.

The evolution of synthetic genomes is likely to provide a new realm of study in evolutionary biology. The observation of vector and parasite evolution in response to genetic interventions will provide many new opportunities to study evolution in real time, integrated with genetics, ecology and life history. When massive population suppression is achieved, we may even observe the evolution of novel escapes that provide insight to the ‘macroevolution’ of genetic systems. The effort will be one of experimental evolution on a huge scale in a realistic context, allowing us to revisit classic problems in life history evolution [100] and genetic systems [101, 102].
